# Metastasis of breast cancer to bones alters the tumor immune microenvironment

**DOI:** 10.1186/s40001-023-01083-w

**Published:** 2023-03-13

**Authors:** Xue Chao, Ying Zhang, Chengyou Zheng, Qitao Huang, Jiabin Lu, Emilia M. Pulver, Julia Houthuijzen, Stefan Hutten, Rongzhen Luo, Jiehua He, Peng Sun

**Affiliations:** 1 State Key Laboratory of Oncology in South China, Collaborative Innovation Center for Cancer Medicine, Sun Yat-sen University Cancer Center, Guangzhou, China; 2 Department of Pathology, Sun Yat-sen University Cancer Center, Sun Yat-sen University, 651 Dongfeng East Road, Guangzhou, 510120 China; 3 Division of Molecular Pathology, The Netherlands Cancer Institute, 1066 CX Amsterdam, The Netherlands

## Abstract

**Background:**

Bone is one of the most frequent sites for breast cancer metastasis. Breast cancer bone metastasis (BCBM) leads to skeletal morbidities including pain, fractures, and spinal compression, all of which severely impact quality of life. Immunotherapy is a promising therapy for patients with advanced cancer, but whether it may provide benefit to metastatic bone cancer is currently unknown. Thus, a better understanding of the immune landscape of bone-disseminated breast cancers may reveal new therapeutic strategies. In this study, we use histopathological analysis to investigate changes within the immune microenvironment of primary breast cancer and paired BCBM.

**Methods:**

Sixty-three patients with BCBM, including 31 with paired primary and bone metastatic lesions, were included in our study. The percentage of stroma and stromal tumor-infiltrating lymphocytes (TILs) was evaluated by histopathological analysis. The quantification of stromal TILs (CD4 + and CD8 +), macrophages (CD68 + and HLA-DR +), programmed cell death protein 1 (PD-1), and programmed cell death protein ligand 1 (PD-L1) was evaluated through immunohistochemical (IHC) staining. Statistical analysis was performed with paired *t* test, Wilcoxon test, spearman correlation test, and univariate and multivariate cox regression.

**Results:**

Median survival after BCBM pathological diagnosis was 20.5 months (range: 3–95 months). Of the immune parameters measured, none correlated with survival after bone metastasis was diagnosed. Compared to the primary site, bone metastases exhibited more tumor stroma (mean: 58.5% *vs* 28.87%, *p* < 0.001) and less TILs (mean: 8.45% *vs* 14.03%, *p* = 0.042), as determined by H&E analysis. The quantification of primary vs metastatic tissue area with CD4 + (23.95/mm^2^ *vs* 51.69/mm^2^, *p* = 0.027 and with CD8 + (18.15/mm^2^ *vs* 58.95/mm^2^, *p* = 0.004) TILs similarly followed this trend and was reduced in number for bone metastases. The number of CD68 + and HLA-DR + macrophages showed no significant difference between primary sites and bone metastases. PD-1 expression was present in 68.25% of the bone metastasis, while PD-L1 expression was only present in 7.94% of the bone metastasis.

**Conclusions:**

Our findings suggest that compared to the primary breast cancer site, bone metastases harbor a less active immune microenvironment. Despite this relatively dampened immune landscape, expression of PD-1 and PD-L1 in the bone metastasis indicates a potential benefit from immune checkpoint inhibitors for some BCBM cases.

**Supplementary Information:**

The online version contains supplementary material available at 10.1186/s40001-023-01083-w.

## Background

Bone metastasis occurs in 70% of patients with advanced breast cancer [1]. The skeletal-related events (SREs) associated with breast cancer bone metastasis (BCBM), such as pathological fracture, spinal cord compression, and severe pain, impair the patient’s mobility, reduce their quality of life, and increase overall morbidity [2]. Metastasis to the bone means that tumor cells leave their primary site and migrate to a new and specialized microenvironment made up of osteoblasts, osteocytes, osteoclasts, adipocytes, hematopoietic stem cells, and immune cells [3].

Current primary treatment options for BCBM include radiotherapy, osteoclasts inhibitors, endocrine therapy, chemotherapy, and supportive treatment-like analgesia. Occasionally, surgery can be applied when a single metastasis is found or when acute spinal injury happens. However, even with these options and multidisciplinary approaches, the metastases eventually develop resistance and progress. Increasing evidence suggests that immunotherapy may be a promising treatment method for primary breast cancers. Tumor-infiltrating lymphocytes (TILs) are reported to correlate with survival and therapeutic efficacy in breast cancer, especially in triple-negative and HER2 positive breast cancer subtypes [4–7]. Moreover, another study demonstrated that TILs at metastatic sites of breast cancer also correlate with improved survival [8, 9].

The programmed cell death protein 1 (PD-1)/programmed cell death ligand 1 (PD-L1) pathway is an immune checkpoint pathway that suppresses immune system activation, where binding of the T cell receptor PD-1 to its ligand PD-L1 leads to downregulation of T cell proliferation, activation, and host anti-tumor function [10]. Tumor cells can exploit this checkpoint by expressing PD-L1 and, therefore, evade anti-tumor immune responses. PD-L1 expression on primary breast tumor cells, however, can be targeted with immune checkpoint inhibitors (ICIs) and subsequently correlates with immunotherapeutic benefit in the clinic and improved patient survival [11]. Moreover, ICIs have proven effective in all molecular subtypes of breast cancer [12–14].

Despite this clinical advance in treating primary breast cancers with ICIs, limited data are available on the role of the immune microenvironment of BCBM and its impact on treatment responsiveness. A better understanding of tumor cell bone residence and interaction with the immune microenvironment may, therefore, unveil new targetable vulnerabilities and guide clinically relevant therapeutic approaches. This study aims to investigate the immune microenvironment shift between primary tumor sites and bone metastases of breast cancer by evaluating TILs, macrophages, and immune checkpoint markers.

## Materials and methods

### Study population and data collection

This study was conducted using data from patients treated at the Sun Yat-sen University Cancer Center who underwent a breast cancer bone metastasis (BCBM) biopsy or excision between January 2017 and August 2020. Patients with a previous history of malignant tumor and immune deficiencies were excluded. The clinical parameters used in this investigation were obtained from original medical records which included age, pathological diagnosis, symptoms, present and past medical history, image examination including ultrasound and mammography results, operative records, and adjuvant therapy data. The follow-up information was collected from medical records and by telephone interviews. The primary endpoint of the study was disease progression-free survival. This study was conducted in accordance with the ethical standards of the research committee of SYSUCC (IRB number: B2021-076-01). The SYSUCC ethical committee exempted the informed consent of this study.

### Pathological assessment

All tumor sections from metastatic sites were reviewed independently by two pathologists, as well as the matched primary tissue when available. The estrogen and progesterone hormone receptors and HER2 receptor status were gathered from the original pathological reports.

### Stroma evaluation

Stroma percentage was evaluated following the Mesker’s study protocol [15] using one representative H&E slide digital scan per patient case. Scoring percentage was given in a 10% fold. Two pathologists evaluated the data independently while blinded to the clinical outcome. Consensus was reached between the two pathologists if there was a discrepancy among the collected scored data.

### TILs evaluation

TILs percentage was counted both manually and automatically according to the system developed by the International Immuno-oncology Biomarker Working Group [16, 17]. This method was described before [18]. In addition to the automatic software quantification, two pathologists independently evaluated the data and were blinded to the clinical outcome. Consensus was reached between the two pathologists if there was a discrepancy among the collected scoring.

### Immunohistochemical evaluations

Formalin-fixed paraffin-embedded (FFPE) tissue sections were IHC stained for PD-1 (Clone UMAB199, ZSGB-Bio), PD-L1 (Clone SP142, Roche Diagnostics), CD4 (Clone EP204, ZSGB-Bio), CD8 (Clone SP16, ZSGB-Bio), CD68 (Clone PG-M1, ZSGB-Bio), and HLA-DR (EPR3692, Abcam). Due to the small size of lymphocytes, an immune cell was considered ‘PD-L1/PD-1-positive’ if it featured any PD-L1 staining. Membranous or cytoplasmic expression of PD-1 or PD-L1 in immune cells that was ≥ 1% was considered positive expression. Quantification of CD4 + and CD8 + TILs and CD68 + and HLA-DR + macrophages by area was performed manually by two pathologists through digital scan of the slides. Consensus was reached between the two pathologists if there was a discrepancy among the collected data.

### Statistics

Categorical variables were grouped based on the clinical findings, and decisions on the groups were made before modeling. The results were compared using the *χ*^2^ test or Fisher’s exact test. Continuous variables were compared using the *t* test. Spearman’s rank correlation tests were used to assess the association. A *p* value of < 0.05 was considered statistically significant. All statistical analyses were carried out using the SPSS software, version 25.0 (IBM Corp, 1987, Chicago, USA) and GraphPad Prism 8 (GraphPad software, Inc).

## Results

### Patients’ characteristics

Sixty-three patients with BCBM, of which 31 included paired primary lesion with bone metastasis, were included in our study. Of these primary breast tumors, 63.4% (*n* = 40) of patients had luminal type tumors, 23.8% (*n* = 15) had HER2 amplification, 6.34% (*n* = 4) were triple-negative subtype, and 6.34% (*n* = 4) were classified as uncertain. The majority of tumors were ductal rather than lobular, where 51 (80.9%) patients had invasive ductal carcinoma of non-special type. Five (7.93%) patients were initially diagnosed with bone metastasis of breast cancer, and they did not receive radical surgery. Forty (63.49%) patients developed bone metastasis as the first progression site. The median age at bone metastasis was 43 years (range: 25–71 years) and the median time to bone metastasis from first diagnosis was 38 months (range: 0–204 months). The most common site of BCBM was spinal vertebra (*n* = 43, 68.25%). The median survival after BCBM was pathologically diagnosed was 20.5 months (range: 3–95 months). Details are listed in Table 1.

**Table 1:** Patient characteristics

Variable	*N*	%
Bone metastasis lesions	63	
Primary tumor and matched bone metastasis	31	49.21
Primary tumor histology		
Ductal, non-special type	51	80.95
Ductal, micropapillary	1	1.59
Ductal, mucinous	2	3.17
Ductal and lobular	2	3.17
Uncertain	7	11.11
Primary tumor grade		
1	1	1.59
2	15	23.81
3	40	63.49
Uncertain	7	11.11
Primary tumor phenotypes		
Luminal A	11	17.46
Luminal B	29	46.03
HER2 amplification	15	23.81
Triple-negative	4	6.35
Uncertain	4	6.35
Primary tumor size		
< 2 cm	11	17.46
2–5 cm	24	38.10
> 5 cm	2	3.17
Uncertain	26	41.27
Nodes status		
Negative	10	15.87
1–3 nodes	20	31.75
4–9 nodes	4	6.35
> 10	13	20.63
Uncertain	16	25.40
Surgery		
Mastectomy	44	69.84
Breast conserving surgery	5	7.94
None	5	7.94
Uncertain	9	14.29
Chemotherapy		
Adjuvant	54	85.71
Neoadjuvant	1	1.59
None	8	12.70
Radiotherapy		
Adjuvant	26	41.27
None	37	58.73
Trastuzumab/pertuzumab application in HER2-positive patients	7/15	
Endocrine therapy		
Yes	40	63.49
No	23	36.51
Bone metastasis at first diagnosis	5	7.94
Bone metastasis sites		
Ilium	5	7.94
Sternum	7	11.11
Rib	1	1.59
Vertebra	43	68.25
Femur	3	4.76
Humerus	1	1.59
Skull	1	1.59
Treatment after bone metastasis		
Radiotherapy	8	12.70
Chemotherapy	27	42.86
Endocrine therapy	42	66.67
Trastuzumab/pertuzumab	4	6.35
Alive at last follow-up	43	68.25

### BCBM microenvironment characteristics

The median stromal TILs percentage in BCBM was 5% (interquartile range [IQR]: 5–10%). The BCBM microenvironment contained similar numbers of CD4 + (median: 12.5/mm^2^; IQR: 1–27.5/mm^2^) and CD8 + (median: 5/mm^2^; IQR: 1–35/mm^2^) TIL subpopulations. Both the CD4 + and the CD8 + TIL infiltrates correlated with overall BCBM TILs percentage (*r* = 0.32, *p* = 0.01 and *r* = 0.33, *p* < 0.01). HLA-DR + macrophages (median: 85/mm^2^; IQR: 60–120/mm^2^) dominated in the macrophage subtypes present; stromal CD68 + were far less prevalent (median: 37.5/mm^2^; IQR: 12.5–57.5/mm^2^). PD-1 expression was found in 68.25% of patient BCBMs, while PD-L1 expression was found in only 7.94% (Table 2). No significant difference in BCBM tumor immune parameters was found between the different primary molecular subtypes (Additional file 1: Fig S1).

**Table 2:** Assessment of tumor microenvironment of breast cancer bone metastasis

Variable	Median	IQR
Stroma (%)	70	40–80
Stromal TILs (%)	5	5–10
Stromal CD4 + TILs (/mm^2^)	12.5	1–27.5
Stromal CD8 + TILs (/mm^2^)	5	1–35
CD4/CD8 ratio	1	0.53–5.18
Stromal HLA-DR + macrophages (/mm^2^)	85	60–120
Stromal CD68 + macrophages (/mm^2^)	37.5	12.5–57.5
HLA-DR/CD68 ratio	2.27	1.44–5
PD-1 expression	43/63	68.25 (%)
PD-L1 expression	5/63	7.94 (%)
Osteoclasts (/mm^2^)	0.33	0–3

At the time that all 63 samples were collected, 9 (14.29%) patients were undergoing chemotherapy, 1 (1.58%) was under local breast radiotherapy, 25 (39.68%) were receiving endocrine therapy, and the remaining 28 patients (44.44%) were without any current therapy. We analyzed the immune microenvironment parameters based on these treatment groups (Additional file 2: Fig S2). In general, there was no significant difference between the treatment groups; however, we observed a trend of decreasing CD8 + TILs in patients treated with chemotherapy but this trend was not statistically significant (*p* = 0.19).

### Comparison between primary site and BCBM

#### Stroma percentage

**Fig. 1:**
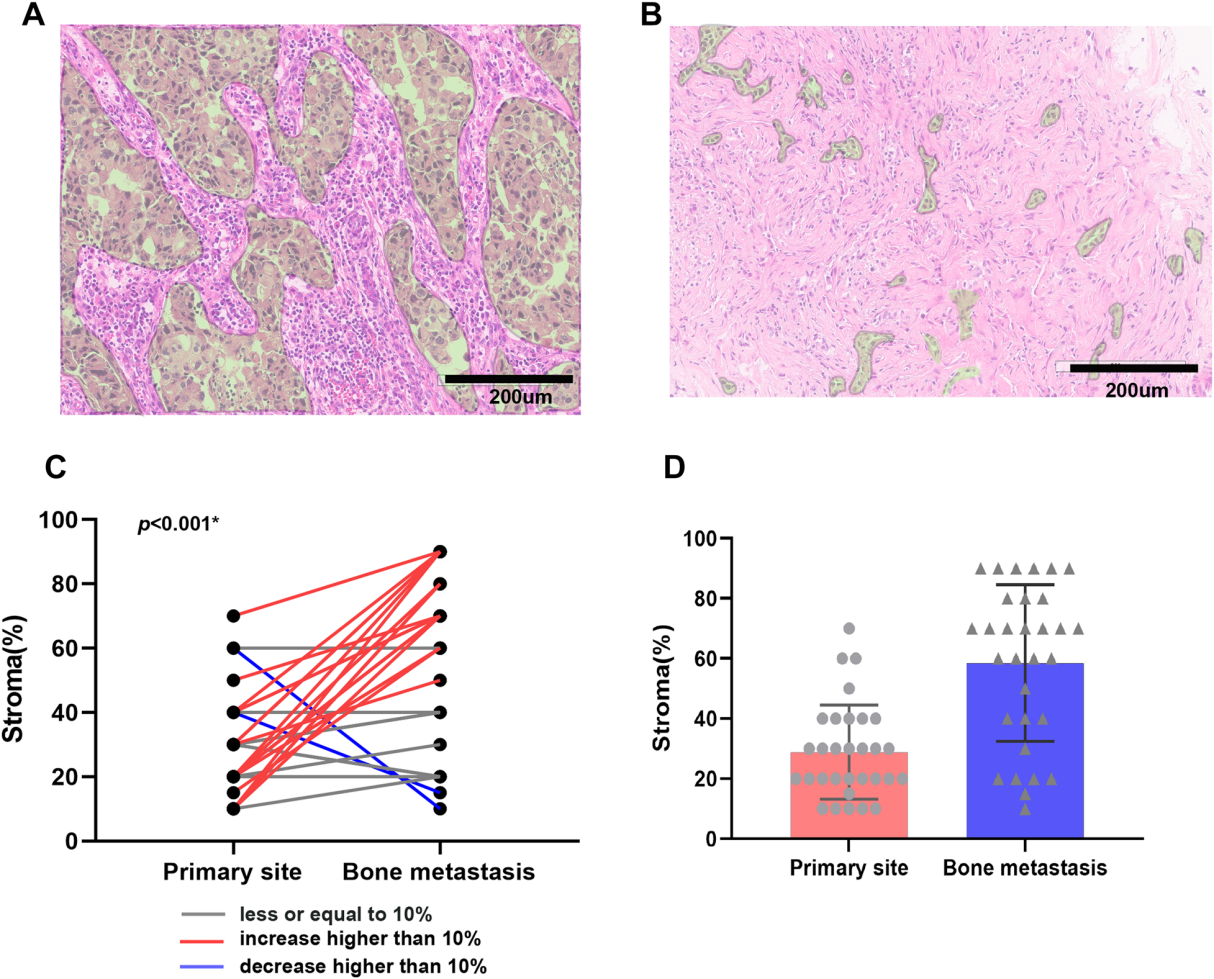
Example of paired primary site **A** and bone metastasis site **B** with the staining of Hematoxylin&Eosin (Original magnification × 200) showed increasing stroma percentage in the bone metastasis compared with the primary site. The segmented area in green indicates the tumor compartment. The rest were stroma compartment. Change of stroma percentage trend **C** and value **D** between primary site and bone metastasis

Accounting for all molecular subtypes, the bone metastasis site contained more stroma compared to its primary site (mean: 58.5% *vs* 28.87%, *p* < 0.001; Fig. 1). The luminal type breast cancer which made up the majority of patient samples specifically followed this trend (mean: 63.42% *vs* 28.42%, *p* < 0.001; Additional file 3: Fig S3).

#### Comparison of primary and bone metastatic immune microenvironment

**Fig. 2:**
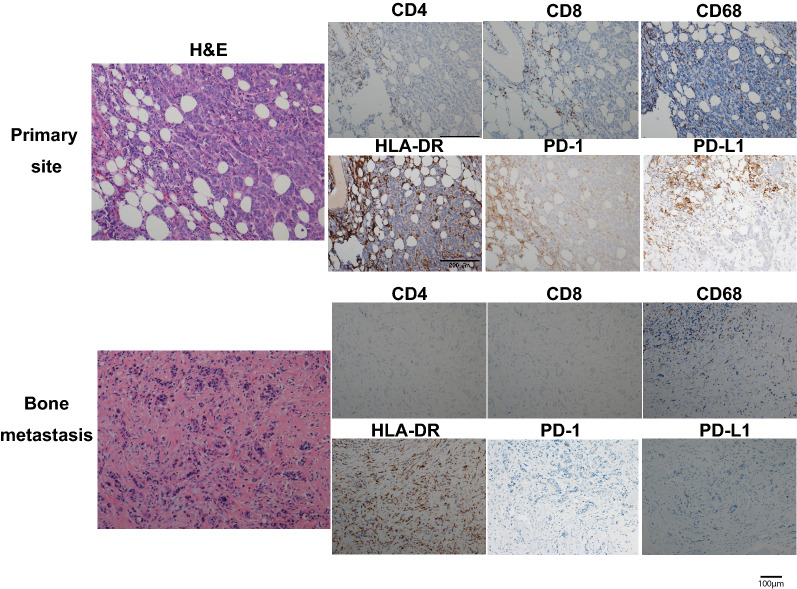
Example of paired primary site and bone metastasis site with the staining of Hematoxylin&Eosin, CD4, CD8, CD68, HLA-DR, PD-1 and PD-L1. (Original magnification × 200)

**Fig. 3:**
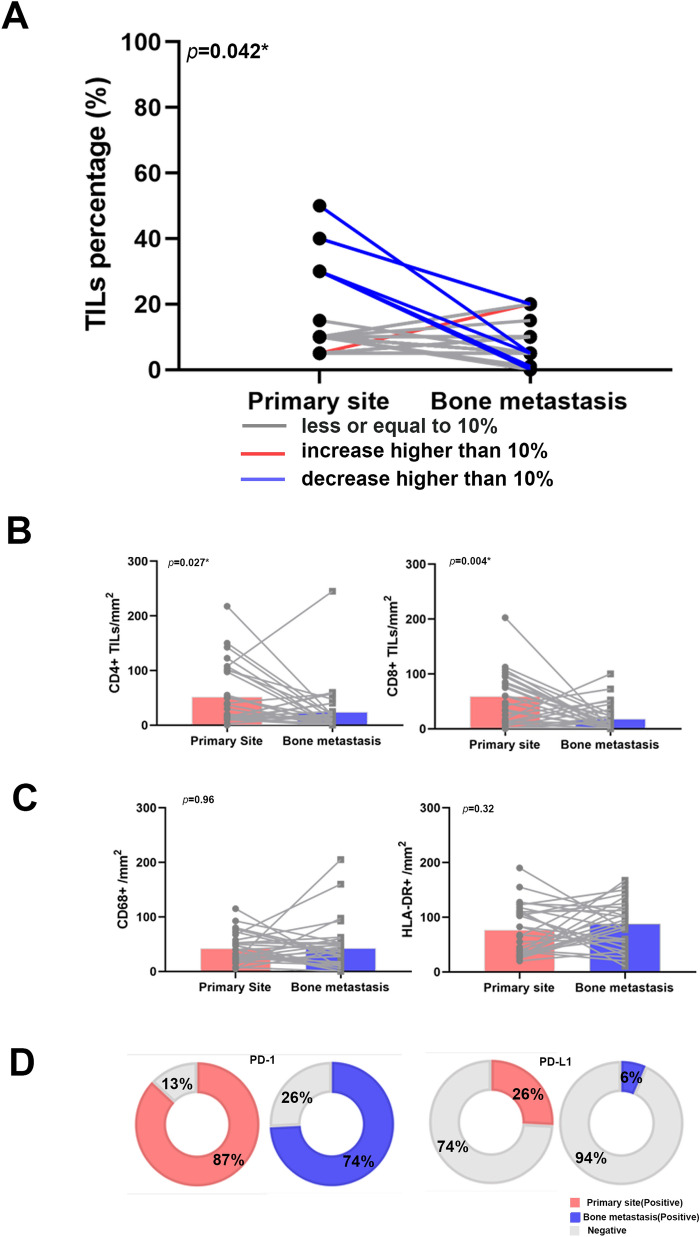
Stromal TILs counts in primary site and bone metastasis. **A** Change in TILs percentage overall. **B** CD4 + and CD8 + TILs counts in primary site and bone metastasis. **C** CD68 + and HLA-DR + Macrophages counts in primary site and bone metastasis. **D** PD-1 and PD-L1 positivity percentage in primary site and bone metastasis. The asterisk in the figure refers to *p* < 0.05

**Fig. 4:**
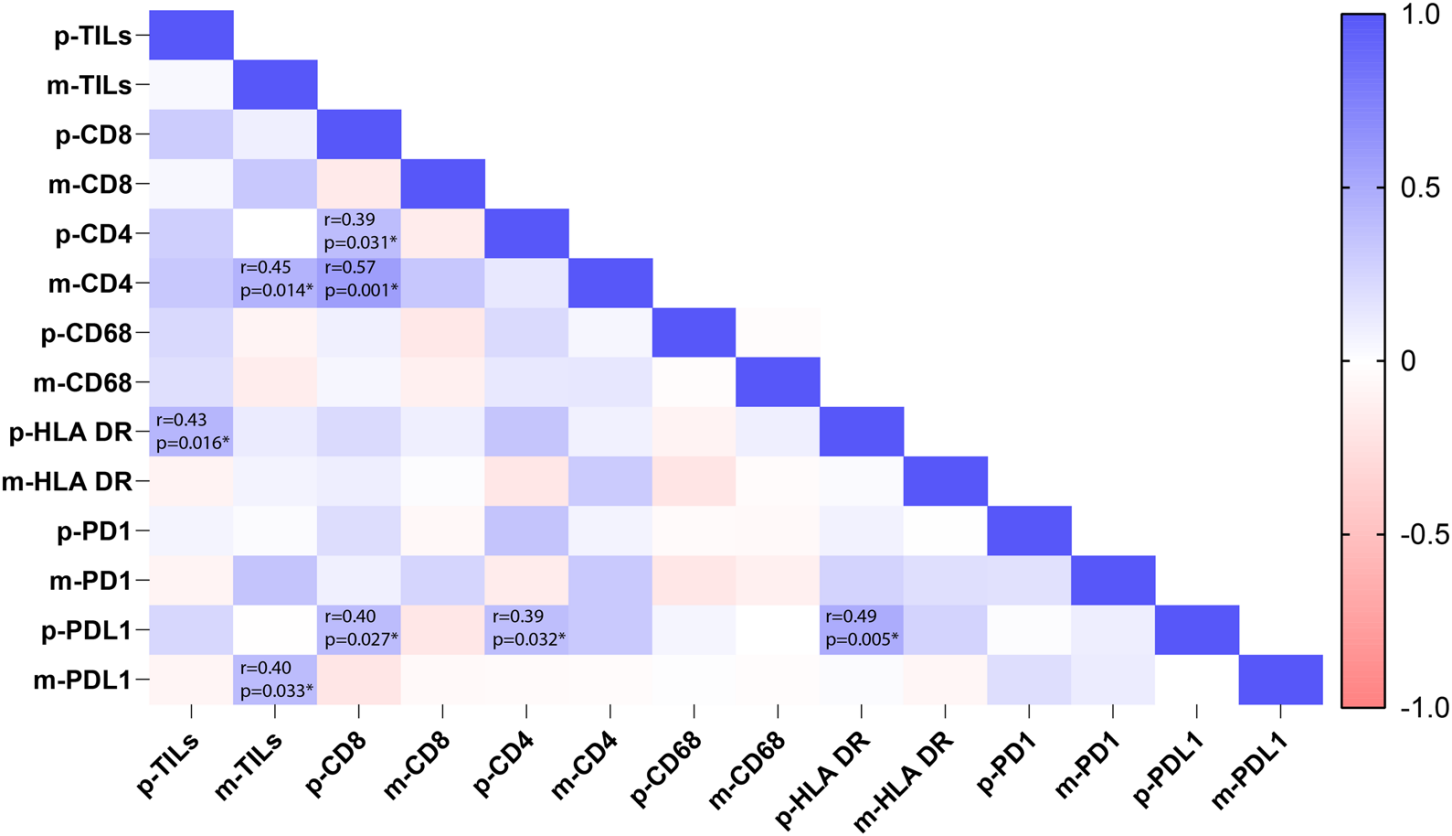
Correlation matrix of immune parameters between primary site and bone metastasis. The asterisk in the figure refers to *p* < 0.05

Thirty-one paired primary breast tumor and bone metastasis cases were analyzed via IHC (Fig. 2). Compared to the primary site, bone metastases displayed less overall TILs (mean: 8.45% vs 14.03%, *p* = 0.042; Fig. 3A). We also compared specific TILs subpopulations, macrophages, and immune checkpoint molecules between primary and metastatic sites. CD4 + TILs and CD8 + TILs at BCBM sites were likewise decreased in number compared to primary tumors (Fig. 3B). No significant difference was observed in the CD4 + /CD8 + ratio. Furthermore, both CD68 + and HLA-DR + macrophage types showed no significant difference in expression between primary site and bone metastasis site, or in relation to each other (Fig. 3C). The PD-1 and PD-L1 positive rate in bone metastasis also decreased compared with primary site (Fig. 3D). Analysis based on molecular subtype is displayed in Additional file 4: Fig S4. All immune parameters of primary and the metastatic sites were combined to input a correlation matrix (Fig. 4).

## Discussion

We did a comprehensive histopathological analysis of the BCBM microenvironment. This is also the largest study to date to investigate immune microenvironment differences between primary breast cancer and its bone metastases. We observed that bone metastasis has a different distribution of stromal compartment and has a less active immune compartment compared with the primary disease site.

Stroma percentage in primary breast tumors is a proven prognostic factor; higher stroma percentage often correlates with increasing relapse rate and poorer long-term survival [19–21]. Given this reported correlation and considering metastatic diseases are inherently advanced in nature with a likely worse prognosis, the increased percentage of stromal tissue found in the bone metastasis compared to the primary tumor site may not be surprising. However, we did not observe a significant correlation between stroma percentage of primary tumors or of the bone metastasis sites with survival. This may be due to the limited sample size of this study, or may point to other unique aspects of BCBM that influence and modulate disease progression.

Osteoclasts are the main participant in bone remodeling by secreting acid and lytic enzymes and modulating osteolytic processes [22]. Most BCBMs are osteolytic lesions. Osteoclasts display an outstanding morphological characteristic—a large multinucleate bone cell—which makes it easy to identify by microscopy [23]. In our study, we observed that all 63 BCBM lesions presented a prominent osteolytic change through the slides. Thirty-eight (60.32%) of the lesions presented osteoclasts around the cancer cells. These findings suggest that treatment targeted at osteoclasts could be effective in these patients.

Immune cells may play a crucial role in supporting bone metastasis and also have a relevant relationship with osteoclasts. Osteoclasts are derived from progenitor cells, which can also differentiate into macrophages and lymphocytes. Second, the receptor activator of nuclear factor-ƙB ligand (RANKL), which works as major regulator of osteoclasts, can be produced by immune cells [24]. In addition, bone marrow is a place, where tumor cells can direct contact with the immune system [25]. We observed that, compared to the primary site, bone metastasis sites had a less active immune environment, especially when considering TILs (Fig. 3). Studies which compared different metastatic sites also revealed that metastatic breast cancers are immunologically more inert than their corresponding primary tumors [26, 27]. Previous studies also showed that TILs correlate with survival in metastatic breast cancer sites [8, 9]. We did not observe this trend in BCBM. The luminal type breast cancer accounts for approximately 60% of the BCBMs [28, 29]. A reason for this could be that 40/63 (63.49%) of our patients had luminal type breast cancers, a molecular subtype with a prognosis that is less associated with TILs expression [4], and these luminal subtypes in our cohort showed less TILs in metastatic sites compared to the other molecular subtypes [30]. The sample size of HER2-positive and triple-negative patients were too limited to draw a conclusion that may otherwise have supported this trend. Studies on breast cancer metastasis to the lung, liver, and brain reported that high TILs correlated with better survival in triple-negative patients [27, 30]. CD4 + regulatory T cells are a known source of RANKL-induced metastases [26]. CD8 + T cells, also called cytotoxic T cells, however, are the main actor in the anti-cancer immune system that inhibits metastasis [27]. We observed a decrease in both CD4 + T cells and CD8 + T cells in bone metastasis compared with primary sites and without a significant change in the CD4 + /CD8 + ratio. This decrease aligns with a pro-metastasis tumor environment that can support BCBM, but their presence may nonetheless offer opportunities to apply certain immunotherapies and warrants further investigation.

Based on the correlation analysis we did of primary and metastasis site. We did not observer any significant correlation between primary and metastatic sites. Timepoints for the sampling collection, sampling site, and therapy regimes may possibly confound these results. We found that PD-L1 expression correlateswith TILs, CD4, CD8 especially in primary site, which is as expected. Previous clinical trials also showed that PD-L1 expression correlates with TILs and the response [31-33]. A combination of TILs and PD-L1 expression evaluation to select optimal patients for immunotherapy could be a better approach [34].

In the past few years, immunotherapy has become a promising therapy for late-stage breast cancer patients. Recent studies suggest that combination immunotherapies effectively improved the prognosis and survival of PD-L1 + patients [13, 28, 29]. In our study, 5/63 (7.94%) of patients were PD-L1 + . Three of the PD-L1 + patients had luminal type tumors who may not benefit from the PD-1 inhibitors [30]. Nevertheless, combination immunotherapy in bone metastasis patients is associated with better survival [35]. Thus, certain types of BCBM patients may benefit from the combination immunotherapy.

These results offer initial insights into primary and BCBM immune microenvironment differences and open the discussion for targeting these immune features to improve standard and alternative BCBM treatment methods. However, there are limitations to our study. First, it is a retrospective study with a limited sample size from one medical center. Staining on bone tissue also brings challenges due to technical issues which need optimization for better clarity and accuracy in quantification.

## Conclusions

Our study shows that BCBM sites exhibit an abundance of stromal cells and an inactive immune mircroenvironment compared with paired primary breast tumor sites. Application of combination immunotherapy in select groups of BCBM patients deserves further exploration.

## Supplementary Information

**Additional file 1: Figure S1.** Immune parameters of bone metastasis in Luminal type, HER2 positive type and triple negative type breast cancers.

**Additional file 2: Figure S2.** Immune parameters of bone metastasis grouped according to the treatment at the time of sample collection.

**Additional file 3: Figure S3.** Change of stroma percentage between primary site and bone metastasis in Luminal type, HER2 positive type and triple negative type breast cancers.

**Additional file 4: Figure S4.** Change in TILs percentage overall; CD4+ and CD8+ TILs counts in primary site and bone metastasis; CD68+ and HLA-DR+ Macrophages counts in primary site and bone metastasis; PD-1 and PD-L1 positive percentage in primary site and bone metastasis in Luminal type, HER2 positive type and triple negative type breast cancer.

## Data Availability

The data sets during and/or analysed during the current study are available from the corresponding author on reasonable request.

## Abbreviations

BCBM: Breast cancer bone metastasis
TILs: Tumor infiltrated lymphocytes
PD-1: Programmed death 1
PD-L1: Programmed death ligand 1

## References

[1] Coleman RE (2006). Clinical features of metastatic bone disease and risk of skeletal morbidity. Clin Cancer Res.

[2] Sathiakumar N (2012). Mortality following bone metastasis and skeletal-related events among women with breast cancer: a population-based analysis of US medicare beneficiaries, 1999–2006. Breast Cancer Res Treat.

[3] Hofbauer LC (2021). Novel approaches to target the microenvironment of bone metastasis. Nat Rev Clin Oncol.

[4] Denkert C (2018). Tumour-infiltrating lymphocytes and prognosis in different subtypes of breast cancer: a pooled analysis of 3771 patients treated with neoadjuvant therapy. Lancet Oncol.

[5] Seo AN (2013). Tumour-infiltrating CD8+ lymphocytes as an independent predictive factor for pathological complete response to primary systemic therapy in breast cancer. Br J Cancer.

[6] Chao X (2020). Immune parameters associated with survival in metaplastic breast cancer. Breast Cancer Res.

[7] Adams S (2014). Prognostic value of tumor-infiltrating lymphocytes in triple-negative breast cancers from two phase III randomized adjuvant breast cancer trials: ECOG 2197 and ECOG 1199. J Clin Oncol.

[8] Dieci MV (2018). Immune characterization of breast cancer metastases: prognostic implications. Breast Cancer Res.

[9] Duchnowska R (2016). Immune response in breast cancer brain metastases and their microenvironment: the role of the PD-1/PD-L axis. Breast Cancer Res.

[10] Iwai Y (2017). Cancer immunotherapies targeting the PD-1 signaling pathway. J Biomed Sci.

[11] Huang W (2019). Prognostic and clinicopathological value of PD-L1 expression in primary breast cancer: a meta-analysis. Breast Cancer Res Treat.

[12] Schmid P (2018). Atezolizumab and nab-paclitaxel in advanced triple-negative breast cancer. N Engl J Med.

[13] Cortes J (2020). Pembrolizumab plus chemotherapy versus placebo plus chemotherapy for previously untreated locally recurrent inoperable or metastatic triple-negative breast cancer (KEYNOTE-355): a randomised, placebo-controlled, double-blind, phase 3 clinical trial. Lancet.

[14] Rugo HS (2018). Safety and antitumor activity of pembrolizumab in patients with estrogen receptor-positive/human epidermal growth factor receptor 2-negative advanced breast cancer. Clin Cancer Res.

[15] Mesker WE (2007). The carcinoma-stromal ratio of colon carcinoma is an independent factor for survival compared to lymph node status and tumor stage. Cell Oncol.

[16] Salgado R (2015). The evaluation of tumor-infiltrating lymphocytes (TILs) in breast cancer: recommendations by an International TILs working group 2014. Ann Oncol.

[17] Hendry S (2017). Assessing tumor-infiltrating lymphocytes in solid tumors: a practical review for pathologists and proposal for a standardized method from the international immunooncology biomarkers working group: part 1: assessing the host immune response, TILs in invasive breast carcinoma and ductal carcinoma in situ, metastatic tumor deposits and areas for further research. Adv Anat Pathol.

[18] Sun P (2021). A computational tumor-infiltrating lymphocyte assessment method comparable with visual reporting guidelines for triple-negative breast cancer. EBioMedicine.

[19] Gujam FJ (2014). The relationship between the tumour stroma percentage, clinicopathological characteristics and outcome in patients with operable ductal breast cancer. Br J Cancer.

[20] Dekker TJ (2013). Prognostic significance of the tumor-stroma ratio: validation study in node-negative premenopausal breast cancer patients from the EORTC perioperative chemotherapy (POP) trial (10854). Breast Cancer Res Treat.

[21] Roeke T (2017). The prognostic value of the tumour-stroma ratio in primary operable invasive cancer of the breast: a validation study. Breast Cancer Res Treat.

[22] Boyle WJ, Simonet WS, Lacey DL (2003). Osteoclast differentiation and activation. Nature.

[23] Coleman RE (2020). Bone metastases. Nat Rev Dis Primers.

[24] Kawai T (2006). B and T lymphocytes are the primary sources of RANKL in the bone resorptive lesion of periodontal disease. Am J Pathol.

[25] Brylka LJ, Schinke T (2019). Chemokines in physiological and pathological bone remodeling. Front Immunol.

[26] Tan W (2011). Tumour-infiltrating regulatory T cells stimulate mammary cancer metastasis through RANKL-RANK signalling. Nature.

[27] Joseph R (2021). CD8(+) T cells inhibit metastasis and CXCL4 regulates its function. Br J Cancer.

[28] Schmid P (2020). Atezolizumab plus nab-paclitaxel as first-line treatment for unresectable, locally advanced or metastatic triple-negative breast cancer (IMpassion130): updated efficacy results from a randomised, double-blind, placebo-controlled, phase 3 trial. Lancet Oncol.

[29] Loi S (2019). Pembrolizumab plus trastuzumab in trastuzumab-resistant, advanced, HER2-positive breast cancer (PANACEA): a single-arm, multicentre, phase 1b–2 trial. Lancet Oncol.

[30] Tolaney SM (2020). Effect of eribulin with or without pembrolizumab on progression-free survival for patients with hormone receptor-positive, ERBB2-negative metastatic breast cancer: a randomized clinical trial. JAMA Oncol.

[31] Loi S, et al. Relationship between tumor infiltrating lymphocyte (TIL) levels and response to pembrolizumab (pembro) in metastatic triple-negative breast cancer (mTNBC): results from KEYNOTE-086. Annals of Oncology, 2019;20(3):371–382.

[32] Emens L, Loi S, Rugo H. IMpassion130: efficacy in immune biomarker subgroups from phase III study of atezolizumab+ nab-paclitaxel in patients with treatment-naïve, locally advanced or metastatic TNBC. Cancer Res, 2019;79(4):p. GS1–04

[33] Schmid P, et al. Atezolizumab and nab-paclitaxel in advanced triple-negative breast cancer. New England Journal of Medicine, 2018;379(22): 2108–2121 10.1056/NEJMoa1809615 30345906

[34] Gonzalez‐Ericsson PI, et al. The path to a better biomarker: application of a risk management framework for the implementation of PD‐L1 and TILs as immuno‐oncology biomarkers in breast cancer clinical trials and daily practice. The Journal of pathology, 2020;250(5):667–684. 10.1002/path.5406 32129476

[35] Grover P (2019). Bone metastases treated with immune checkpoint inhibitors: a single center experience. J Clin Oncol.

