# Supplementary figures and images for: Metastasis of breast cancer to bones alters the tumor immune microenvironment
Source: Eur J Med Res. 2023 Mar 13;28:119. doi: 10.1186/s40001-023-01083-w (PMC10012464; doi:10.1186/s40001-023-01083-w)

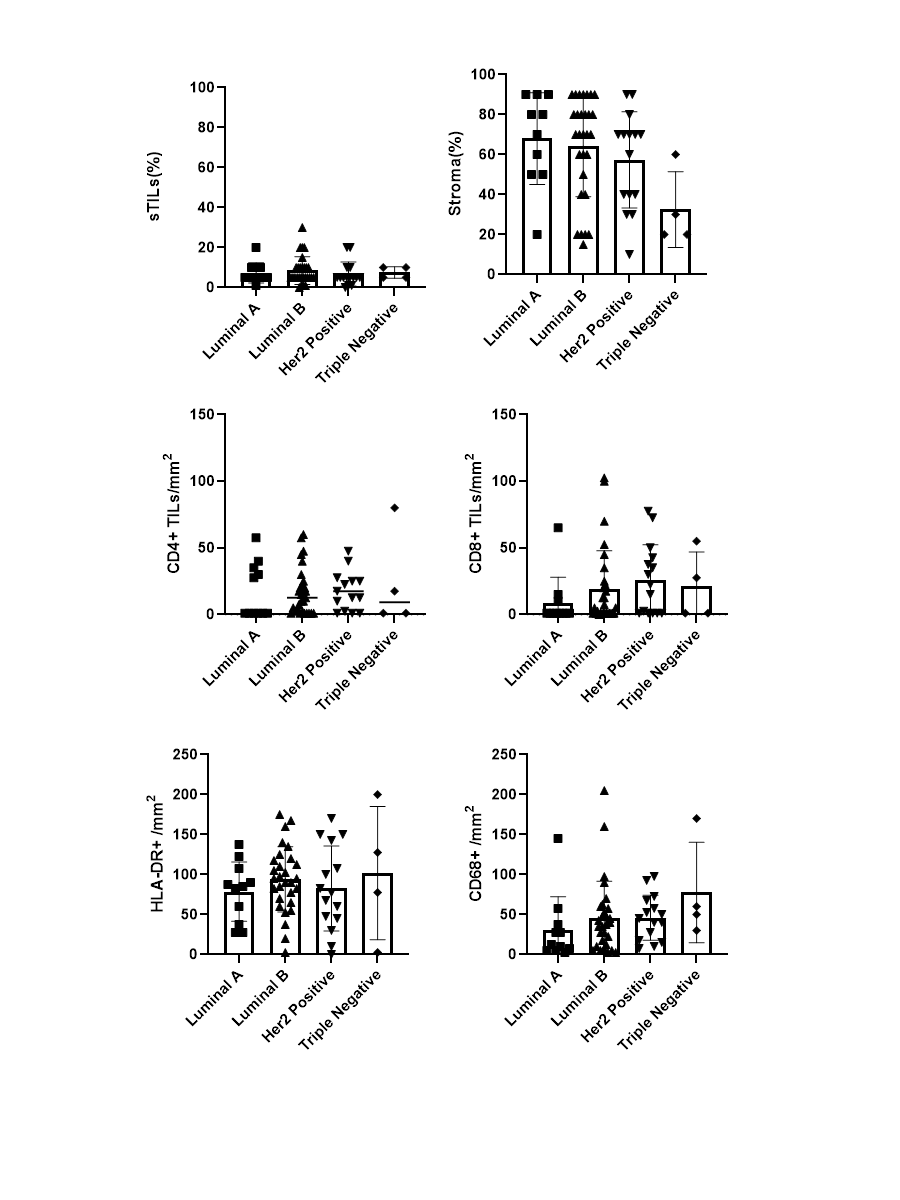

Supplement: Supplementary file 1 — Additional file 1: Figure S1. Immune parameters of bone metastasis in Luminal type, HER2 positive type and triple negative type breast cancers. [file 40001_2023_1083_MOESM1_ESM.tif]

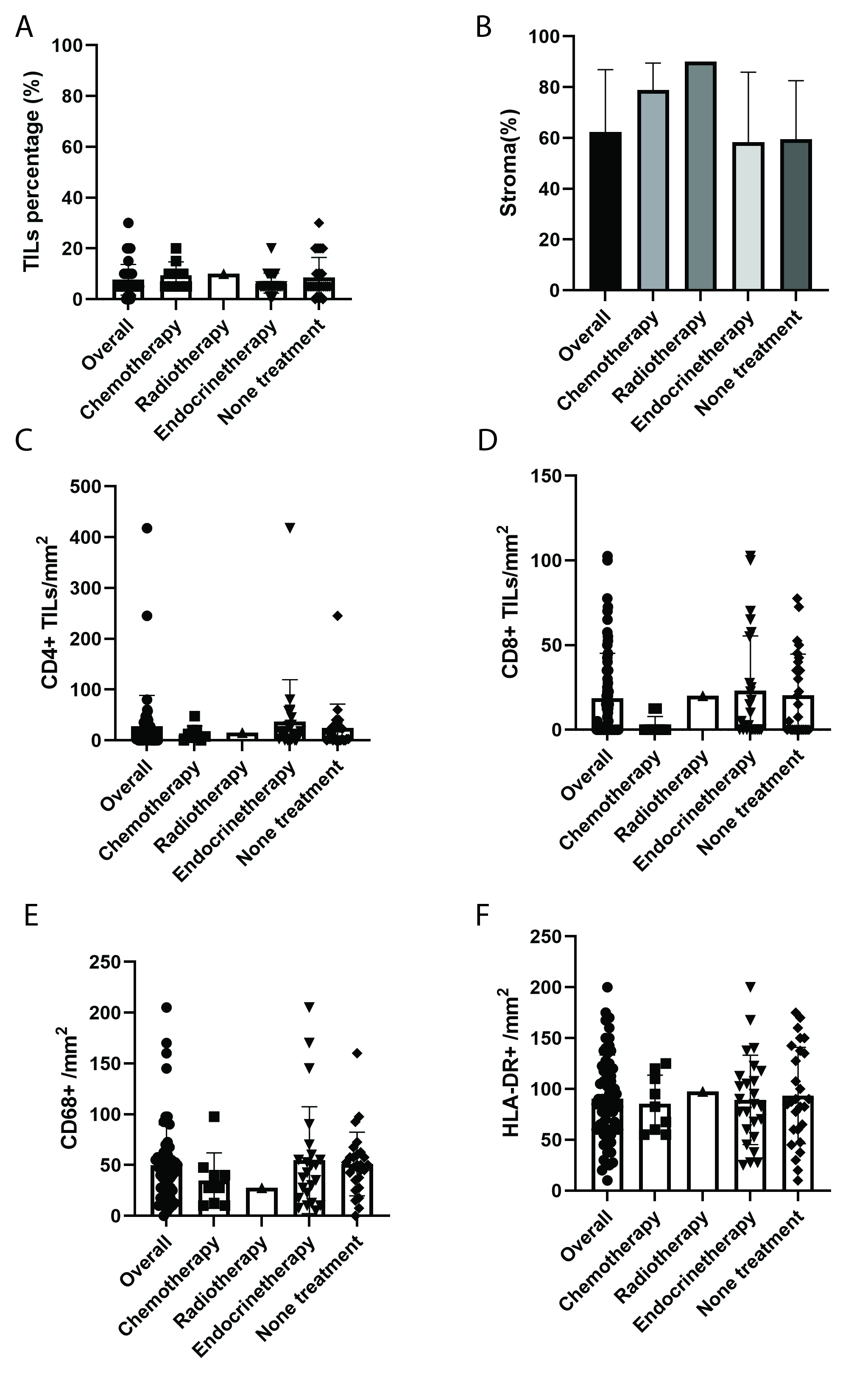

Supplement: Supplementary file 2 — Additional file 2: Figure S2. Immune parameters of bone metastasis grouped according to the treatment at the time of sample collection. [file 40001_2023_1083_MOESM2_ESM.tif]

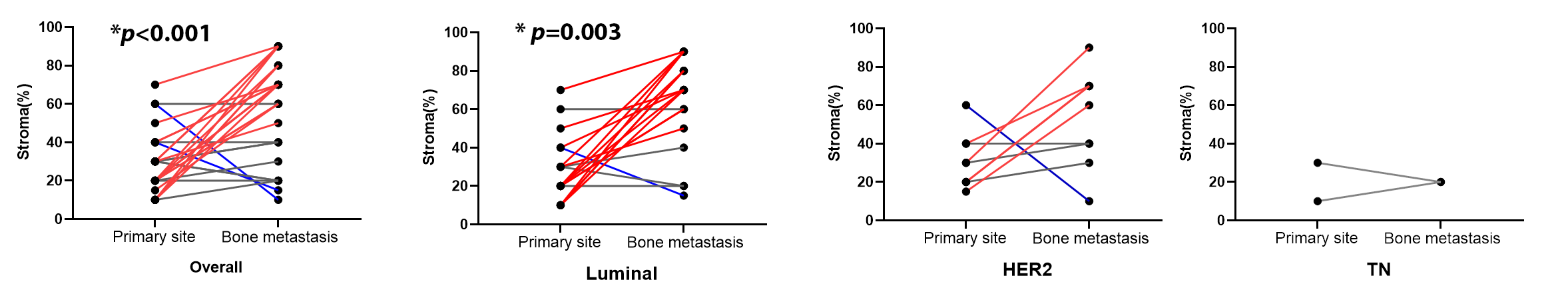

Supplement: Supplementary file 3 — Additional file 3: Figure S3. Change of stroma percentage between primary site and bone metastasis in Luminal type, HER2 positive type and triple negative type breast cancers. [file 40001_2023_1083_MOESM3_ESM.tif]
