# Supplementary material for: Metastasis of breast cancer to bones alters the tumor immune microenvironment
Source: Eur J Med Res. 2023 Mar 13;28:119. doi: 10.1186/s40001-023-01083-w (PMC10012464; doi:10.1186/s40001-023-01083-w)
Supplement: Supplementary file 4 — Additional file 4: Figure S4. Change in TILs percentage overall; CD4+ and CD8+ TILs counts in primary site and bone metastasis; CD68+ and HLA-DR+ Macrophages counts in primary site and bone metastasis; PD-1 and PD-L1 positive percentage in primary site and bone metastasis in Luminal type, HER2 positive type and triple negative type breast cancer. [file 40001_2023_1083_MOESM4_ESM.pdf]

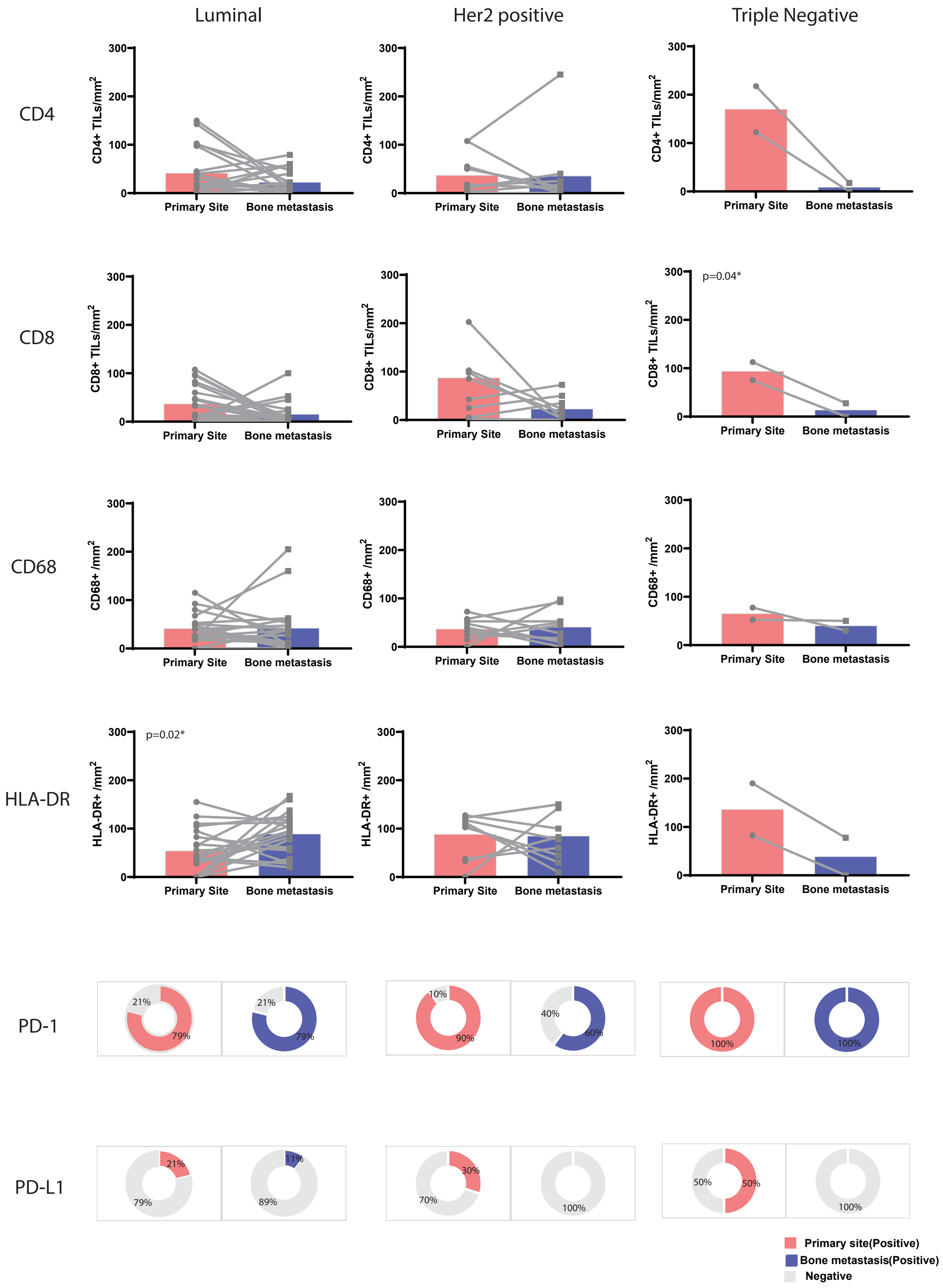

Luminal
Her2 positive
Triple Negative
CD4
CD8
CD68
HLA-DR
PD-1
PD-L1
CD4+ TILs/mm²
CD8+ TILs/mm²
CD68+ /mm²
HLA-DR+ /mm²
Primary Site
Bone metastasis
p=0.04*
p=0.02*
21%
79%
10%
90%
40%
60%
100%
11%
89%
30%
70%
50%
Primary site(Positive)
Bone metastasis(Positive)
Negative
